# An Exploratory Age-Adjusted Model of Individual Alpha Peak Frequency in Healthy Older Adults: A FOOOF-Based Spectral Parametrization Study †

**DOI:** 10.3390/ejihpe16080110

**Published:** 2026-07-28

**Authors:** Rubén Pérez-Elvira, Daniel Cordeiro, María Agudo Juan, Antonio Sánchez Cabaco

**Affiliations:** 1 Faculty of Psychology, Pontifical University of Salamanca, 37002 Salamanca, Spain; asanchezca@upsa.es; 2 Neuropsychophysiology Laboratory, NEPSA Rehabilitación Neurológica, 37003 Salamanca, Spain; mjagudojuan@gmail.com; 3 Computational and Translational Neuroscience Laboratory, 37003 Salamanca, Spain

**Keywords:** individual alpha peak frequency, FOOOF, spectral parameterization, age-adjusted modeling, EEG

## Abstract

Individual alpha peak frequency (iAPF) is a stable neurophysiological marker associated with cognitive processing and healthy brain aging. However, the interpretation of alpha activity in older adults remains limited by the use of fixed canonical frequency bands, which may not adequately capture age-related slowing. The aim of this study was to develop an exploratory age-adjusted model of iAPF in cognitively healthy older adults using FOOOF-based spectral parameterization of resting-state EEG. Eighty-one participants aged 52–87 years were selected from the PDWAVES database. Resting-state, eyes-closed EEG recordings were analyzed over nine parieto-occipital electrodes, and iAPF was defined as the center frequency of the highest alpha peak within the 7–14 Hz range after separating periodic and aperiodic spectral components. Linear regression showed a significant negative association between age and iAPF, described by the equation iAPF = 13.22 − 0.054 × age (F(1,79) = 19.56, *p* < 0.001), indicating an average decrease of 0.54 Hz per decade. Age explained 19.8% of the variance (R^2^ = 0.198), with a mean iAPF of 9.527 ± 1.064 Hz. Bootstrap resampling confirmed that the age–iAPF association was statistically reliable (95% CI for the slope excluding zero), although cross-validation indicated limited predictive performance on held-out data (10-fold mean R^2^ = 0.019; LOOCV R^2^ = 0.155). These findings provide a preliminary age-adjusted reference equation for describing iAPF trajectories in healthy older adults at the group level and support the use of spectral parameterization as a more specific alternative to fixed-band approaches in aging research.

## 1. Introduction

Human electroencephalographic (EEG) activity is characterized by rhythmic oscillations across distinct frequency bands, each associated with specific physiological and cognitive processes (Buzsáki & Draguhn, 2004). The alpha rhythm (8–12 Hz), first described by Hans Berger in 1929 (Berger, 1929), is the most prominent oscillatory component of the resting, eyes-closed EEG over posterior brain regions. Its posterior predominance under eyes-closed conditions and its waxing-and-waning temporal dynamics (i.e., increases and decreases over time) make this activity readily recognizable, such that any activity exhibiting these features could be considered “alpha,” regardless of its nominal frequency range (Amin et al., 2023; Bazanova & Vernon, 2014; Omata et al., 2013). Moreover, this oscillatory activity reflects the synchronization of thalamocortical networks and is linked to relaxed wakefulness, sensory processing, and attentional functions (Hughes & Crunelli, 2005; Jensen & Mazaheri, 2010; Klimesch, 2012).

It is common in the field of neurophysiology to use canonical, a priori frequency bands to label EEG components (e.g., alpha 8–12 Hz, theta 4–8 Hz) (Babiloni et al., 2020). However, reliance on canonical frequency bands may be problematic (Kaiser, 2001; Klimesch, 1999) because frequency bands can exhibit considerable intra-individual variability (Haegens et al., 2014). Indeed, the individual alpha peak frequency (iAPF) has traditionally been used to define the alpha band and, on that basis, to estimate the remaining bands (Babiloni et al., 2020; Klimesch, 1999). The iAPF concept refers to the specific frequency at which each individual shows maximal power within the alpha range (Zhang et al., 2021).

The iAPF is commonly regarded as a stable (“trait-like”) neurophysiological marker that reflects systemic properties of central nervous system function (Grandy et al., 2013; Pérez-Elvira et al., 2021). Empirical work has shown that iAPF is associated with individual differences in cognitive processing speed, working memory performance, and broader information-processing efficiency, with higher iAPF generally linked to faster and better cognitive performance (Klimesch, 1999; Richard Clark et al., 2004). Moreover, iAPF has been related to sensory/perceptual processing, suggesting that individual differences in alpha “pace” may index fundamental constraints on how efficiently sensory information is sampled and processed (Ociepka et al., 2022; Tarasi & Romei, 2024). Moreover, iAPF has been shown to be sensitive to age-related changes in both normal cognitive aging and pathological aging (Grandy et al., 2013; Puttaert et al., 2021).

An age-related decrease in iAPF has been documented (Chiang et al., 2011), which has been linked to changes in cortical network organization and cognitive processing and is also consistent with evidence implicating cholinergic neurotransmission in alpha-range EEG alterations observed with cognitive aging (Schumacher et al., 2020). Precise quantification of this relationship using age-adjusted models is essential for individualized clinical assessment (Leroy et al., 2025).

The iAPF does not constitute merely a descriptor of resting-state EEG, but rather a neurophysiological parameter with intra-individual stability and functional relevance. Available evidence suggests that iAPF shows high temporal stability in both younger and older adults, even in the context of cognitive interventions, supporting its consideration as a trait-like marker rather than a fluctuating state-dependent index. In the same vein, recent test–retest reliability studies have confirmed good-to-excellent levels of consistency for individual alpha peak frequency across different age groups, further reinforcing its utility as a reference variable in the study of healthy brain aging and its possible deviations (Grandy et al., 2013; Popov et al., 2023).

This issue becomes particularly relevant in aging, given that one of the most consistent observations in the literature is the progressive slowing of alpha frequency with age (Scally et al., 2018). However, this slowing is not only of descriptive interest but also has important methodological implications. When the analysis is based on canonical alpha bands defined as 8–12 Hz, part of the dominant oscillatory activity in older adults may be misrepresented or shifted relative to those fixed boundaries (Kaiser, 2001). Consequently, group comparisons or clinical interpretations based on rigid frequency bands may introduce bias and underestimate the true physiological variability associated with aging (Scally et al., 2018).

This limitation is further compounded by the fact that the electrophysiological spectrum is composed of both periodic and aperiodic components. Conventional spectral approaches may conflate genuine changes in alpha oscillatory activity with variations in the aperiodic background of the signal, thereby compromising the physiological interpretation of the results (Donoghue et al., 2020; Tröndle et al., 2023). In this context, spectral parameterization methods such as FOOOF offer a relevant advantage by enabling the separation of oscillatory peaks from the aperiodic component, as well as the estimation of parameters such as center frequency, amplitude, and bandwidth without relying on rigid predefined boundaries (Donoghue et al., 2020). This approach is particularly appropriate in older populations, in whom age-related changes may coexist both in oscillatory activity and in the global organization of the spectrum (Leroy et al., 2025; Merkin et al., 2023).

From a clinical and translational perspective, the availability of age-adjusted references is relevant when a neurophysiological parameter shows systematic variation across aging (Kang et al., 2024; Vermunt et al., 2022). Instead of assuming a single threshold or cutoff, an age-adjusted approach makes it possible to contextualize each individual value according to what would be expected for that age range. Recent studies on resting-state EEG activity have shown age-related changes in both periodic and aperiodic parameters, supporting the need for interpretative frameworks that are sensitive to chronological age. Under this logic, an age-adjusted iAPF model in cognitively healthy older adults may help characterize variability attributable to physiological aging, although distinguishing such variability from pathological deviations will require validation in clinical cohorts (Leroy et al., 2025).

The present study aimed to develop a preliminary age-adjusted model of iAPF in cognitively healthy older adults by means of FOOOF-based spectral parameterization of resting-state EEG. By focusing on posterior regions characterized by predominant alpha activity and separating the periodic and aperiodic components of the spectrum, the study sought to provide a neurophysiologically more precise characterization of the age–iAPF relationship. In line with previous evidence, it was hypothesized that iAPF would show a significant negative association with age, such that an age-adjusted equation could serve as a preliminary reference for describing alpha slowing across healthy aging.

## 2. Materials and Methods

### 2.1. Subjects

Data were accessed through the eBRAIN-Health platform within the eBRAIN-Health project (HORIZON-INFRA-2021-TECH-01-01; Grant Agreement ID: 101058516). The present study used the PDWAVES database metafile available in two sections of the eBRAIN-Health platform (Babiloni et al., 2024a, 2024b) (DOI: 10.25493/8MVP-NPN; DOI: 10.25493/FWHQ-6W5). All available cognitively unimpaired/healthy participants (Nold; n = 81) were included in the analyses (Table 1); no clinical groups were analyzed. Nold exclusion criteria in the source cohort included MMSE < 24, neurological or psychiatric disease, clinically relevant depression (GDS > 9 or Cornell > 10), chronic psychoactive drug use, or significant chronic systemic illness (e.g., diabetes mellitus). Data were pseudonymised prior to sharing, and re-identification keys remained under exclusive control of Sapienza University of Rome.

### 2.2. EEG Recordings and Preprocessing

**Figure 1:**
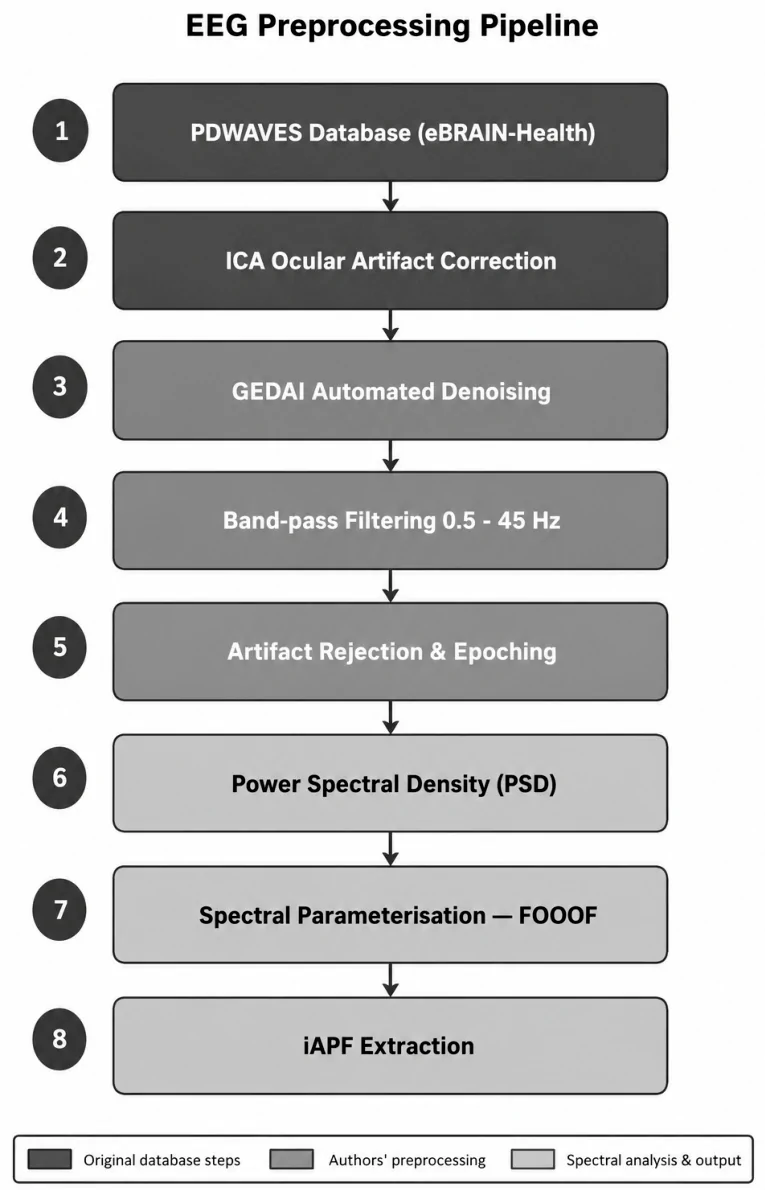
Schematic representation of the EEG preprocessing pipeline. Dark grey boxes indicate steps applied to the original recordings from the PDWAVES database (eBRAIN-Health repository). Mid-grey boxes indicate preprocessing steps implemented in the present study. Light grey boxes correspond to spectral analysis and iAPF extraction. Step 1: resting-state EEG recordings (eyes closed, ~3–5 min). Step 2: ocular artifact correction via Independent Component Analysis (ICA), pre-applied using EEGLAB. Step 3: automated denoising using the GEDAI pipeline. Step 4: band-pass filtering (0.5–45 Hz; FIR filter; electrode impedance < 5 kΩ; sampling rate = 500 Hz). Step 5: artifact rejection (visual inspection and automated peak-to-peak threshold > 100 µV) and segmentation into 2 s epochs. Step 6: power spectral density (PSD) estimation using Welch’s method (2–25 Hz) across nine parieto-occipital channels (Pz, P3, P4, O1, O2, Oz, POz, PO3, PO4). Step 7: spectral parameterisation using the FOOOF algorithm to separate periodic and aperiodic components; goodness-of-fit (R^2^) verified for each participant. Step 8: extraction of the individual alpha peak frequency (iAPF) as the highest spectral peak within the 7–14 Hz range (detection rate: 100%; 81/81 participants). EEG, electroencephalography; ICA, independent component analysis; GEDAI, Guided EEG Denoising with Artifact Identification; PSD, power spectral density; FOOOF, Fitting Oscillations and One-Over-F; iAPF, individual alpha peak frequency.

Resting-state EEG recordings were obtained from the PDWAVES database, available through the eBRAIN-Health repository (Babiloni et al., 2024a, 2024b) (DOI: 10.25493/8MVP-NPN; DOI: 10.25493/FWHQ-6W5). Figure 1 shows the study pipline. EEG acquisition was not performed as part of the present study but followed the original protocol described in the database. Recordings were conducted at Sapienza University of Rome in the late morning, with participants seated in a comfortable armchair in a silent, dimly lit room, instructed to remain awake with eyes closed for approximately 3–5 min.

Prior to their use in the present study, the recordings had undergone an initial preprocessing step as part of the database curation, consisting of ocular artifact correction using independent component analysis (ICA) implemented in EEGLAB. No additional preprocessing had been applied by the data custodians.

For the present analyses, the following additional preprocessing steps were applied by the authors, in this order: (1) automated cleaning using the GEDAI pipeline (Ros et al., 2025) to mitigate residual artifacts; (2) band-pass filtering at 0.5–45 Hz; (3) visual inspection and automated epoch rejection based on a peak-to-peak amplitude threshold (>100 μV); and (4) segmentation into 2 s artifact-free epochs for subsequent spectral parametrization analyses (mean number of retained epochs: 115.4 ± 16.0; range: 76–151; mean recording duration: 3.8 min).

### 2.3. Spectral Parametrization and iAPF Estimation

**Figure 2:**
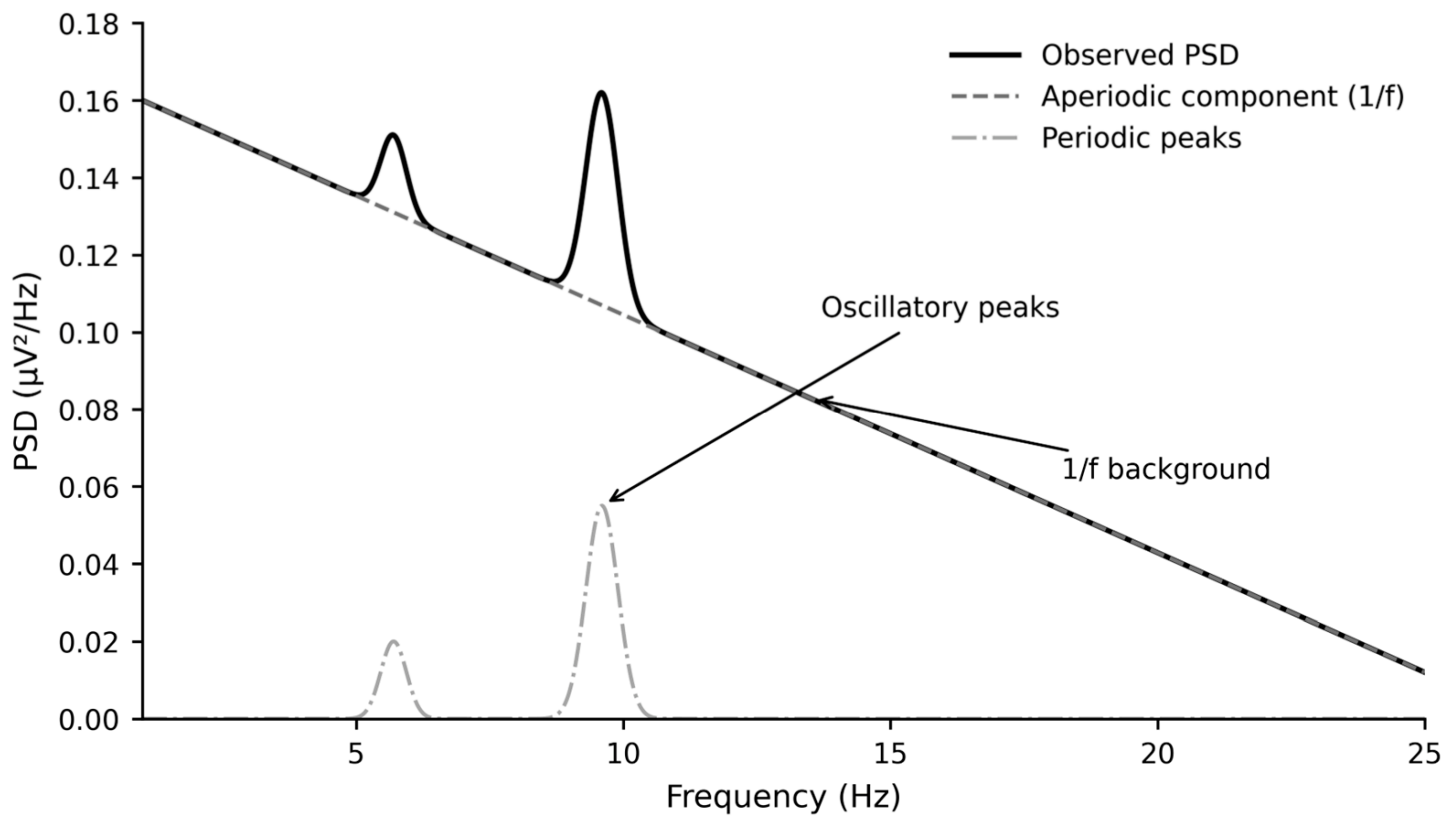
Schematic illustration of spectral parameterization with FOOOF. The observed PSD is modeled as the sum of an aperiodic 1/f-like background and periodic oscillatory peaks. After estimation of the aperiodic component, residual periodic peaks can be identified, and the iAPF is defined as the center frequency of the highest-amplitude peak within the 7–14 Hz range above the fitted aperiodic background.

Power spectral densities (PSDs) were computed over the 2–25 Hz range using Welch’s method (n_fft = 2048, Hann window, 50% overlap, frequency resolution aprox. 0.24 Hz) as implemented in MNE-Python (version 1.12.1). PSDs were then averaged across the available parieto-occipital channels (Pz, P3, P4, O1, O2, Oz; six channels were available across all recordings), where alpha activity is typically maximal. PSDs were parameterized with FOOOF (1.1.0) (Donoghue et al., 2020), which models the spectrum as an aperiodic 1/f component (fixed mode, without a knee inflection) plus a set of Gaussian peaks (Equation (1); Figure 2).

(1) $$P\left(f\right)=\;10^{offset}*\;f^{-exponent}+\;\Sigma_{i}G_{i\left(f\right)}$$

FOOOF was fitted over 2–25 Hz using peak_width_limits = [1, 6] Hz, max_n_peaks = 6, min_peak_height = 0.05, and peak_threshold = 2.0. The aperiodic component was modeled in fixed mode (i.e., without a knee inflection). Goodness-of-fit was assessed using the coefficient of determination (R^2^) and the mean absolute fitting error between the fitted model and the observed PSD. The a priori quality threshold was set at R^2^ > 0.80. Three participants had R^2^ values below this threshold (0.49, 0.70, and 0.74) but were retained after visual inspection confirmed correct alpha peak identification (see Section 3.4). No participants were excluded due to poor spectral fit. The individual alpha peak frequency (iAPF) was defined as the center frequency of the highest-amplitude periodic peak (i.e., above the fitted aperiodic component) within the alpha search range (7–14 Hz) (Figure 2). In addition to the FOOOF-based estimate, a conventional PSD-based iAPF was computed for each participant by identifying the frequency of maximum power within 7–14 Hz on the raw (unparameterized) PSD. The degree of agreement between FOOOF-derived and PSD-derived iAPF estimates was evaluated using Pearson correlation, paired-samples t-tests, and Bland–Altman analysis.

### 2.4. Statistical Analysis

A simple linear regression model was fitted with iAPF as the dependent variable and age as the independent variable. The linearity assumption was tested by comparing the linear model against quadratic, piecewise (segmented) regression, and restricted cubic spline (RCS) alternatives. For the piecewise model, a data-driven breakpoint search was conducted by grid search across the age range (55–84 years) to identify the breakpoint that minimized AIC; the optimal breakpoint was then compared against the a priori candidate of 60 years. RCS models were fitted with 3 and 4 knots placed at recommended percentiles. Model fit was assessed using Akaike Information Criterion (AIC), Bayesian Information Criterion (BIC), and likelihood ratio tests against the linear model. Statistical significance was assessed using the F test (α = 0.05). The coefficient of determination (R^2^), adjusted R^2^, root mean square error (RMSE), and mean absolute error (MAE) were computed as indices of model fit. Model generalization was assessed using 10-fold cross-validation (k = 10) and leave-one-subject-out cross-validation (LOOCV). The 10-fold procedure partitions the sample into 10 subsets of approximately 8 subjects each; given the modest sample size, fold-specific R^2^ values are expected to show high variability due to the small number of subjects per validation subset and the resulting instability of within-fold variance estimates. LOOCV, which uses each participant as a single test case and the remaining 80 as training data, provides a more stable estimate of predictive performance with less susceptibility to fold-composition effects. A permutation test (1000 iterations) was conducted to evaluate whether cross-validated R^2^ values were significantly above chance. Bootstrap resampling with 1000 iterations was used to derive 95% confidence intervals for model parameters, and influential-case diagnostics were performed using Cook’s distance (threshold: D > 4/n). To address the potential covariation between the aperiodic background and iAPF, the aperiodic exponent and offset extracted by FOOOF were examined as predictor variables in exploratory multiple regression models. Pearson correlations were computed between aperiodic parameters and both age and iAPF, and a partial correlation between iAPF and the aperiodic exponent was computed controlling for age. An exploratory multiple regression model (iAPF − Age + Aperiodic Exponent) was fitted and compared to the primary age-only model using AIC and R^2^. All analyses were conducted in Python 3.12.1 using scikit-learn, SciPy, and statsmodels.

## 3. Results

### 3.1. Distribution of the iAPF

**Figure 3:**
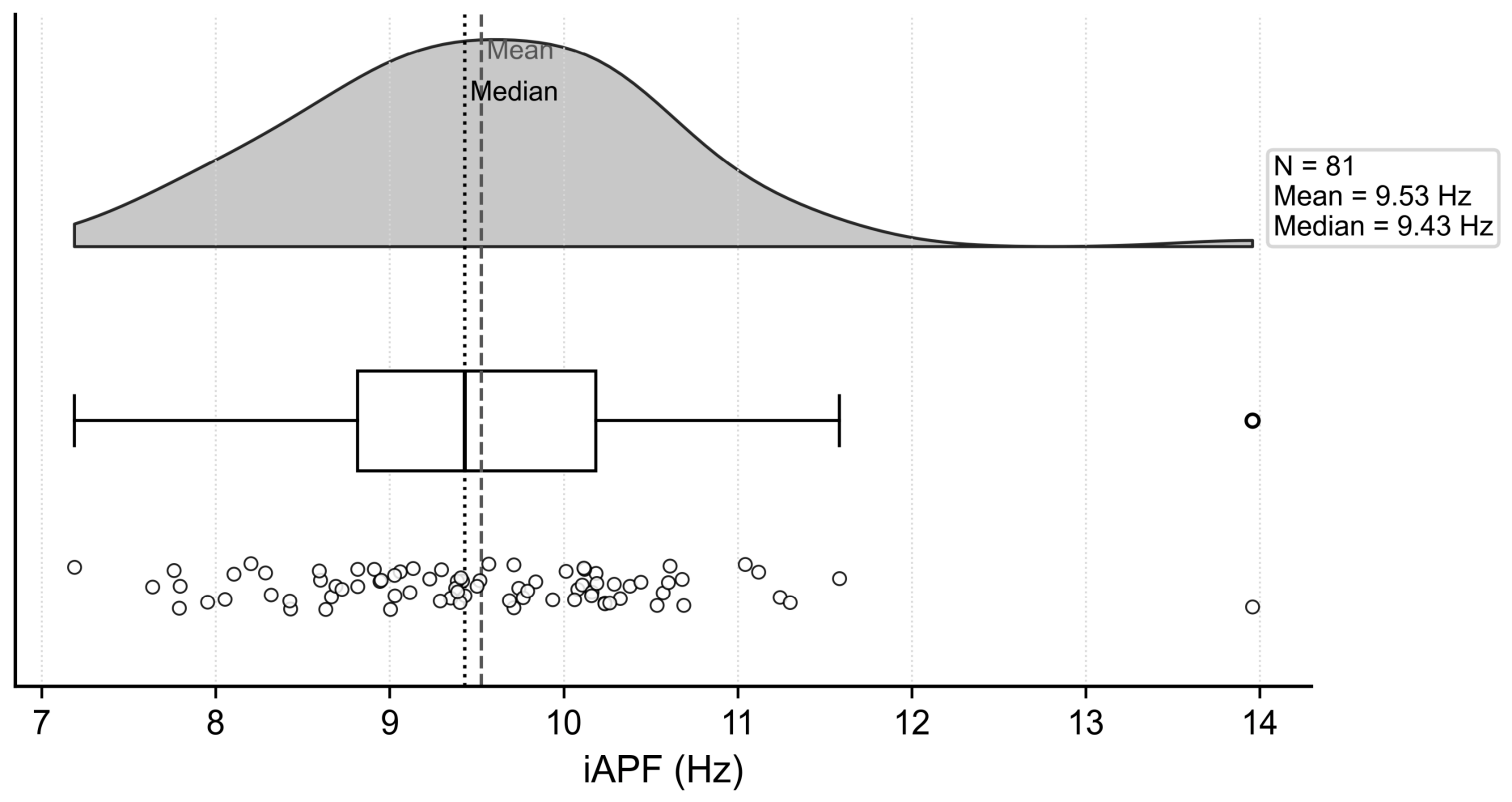
Distribution of individual alpha peak frequency. Raincloud plot of iAPF values in the study sample. The half-violin depicts the data distribution, the boxplot shows the median and interquartile range, and the jittered points correspond to individual participants. Vertical reference lines indicate the mean and median.

The mean iAPF was 9.527 ± 1.064 Hz, ranging from 7.188 to 13.957 Hz. The median was 9.431 Hz. The interquartile range was 8.815–10.185 Hz (Figure 3). FOOOF successfully identified an alpha peak within the 7–14 Hz range in all 81 participants (100% detection rate).

### 3.2. Linear Regression Model

The linear regression model (see Table 2) showed a significant negative relationship between iAPF and age (F(1,79) = 19.56, *p* < 0.001). The model equation was (2):

(2) iAPF (Hz)=13.2165 − 0.0543 × Age (years)

The coefficient of determination was R^2^ = 0.198 (19.8%), indicating that 19.8% of the variability in iAPF is explained by age. The adjusted R^2^ was 0.188, and the residual standard deviation was σ = 0.96 Hz. The RMSE was 0.947 Hz, and the MAE was 0.725 Hz. The negative slope indicates a decrease of approximately 0.54 Hz per decade of age. The correlation between age and iAPF was r = −0.445 (*p* < 0.001).

The linearity assumption was evaluated through model comparison. The linear model yielded the lowest AIC and BIC. The quadratic model did not improve fit (ΔAIC = +2.0; age^2^ coefficient *p* = 0.941), and a piecewise model with a breakpoint at 60 years showed a negligible improvement (ΔAIC = +1.3; likelihood ratio test: *p* = 0.402). A data-driven breakpoint search (grid search across 55–84 years) confirmed that 60 years was the optimal breakpoint, but the resulting model did not significantly outperform the linear specification. Restricted cubic spline models with 3 knots (ΔAIC = +2.0; likelihood ratio test: *p* = 0.951) and 4 knots (ΔAIC = +3.7) likewise failed to demonstrate improved fit. The full set of model comparisons is presented in Table 3. These results indicate that more complex models did not provide clear evidence of improved fit within the present sample. Accordingly, the linear model was retained on grounds of parsimony, while acknowledging that the available evidence supports the adequacy of the linear specification rather than definitively confirming an underlying linear relationship.

### 3.3. Model Validation

Ten-fold cross-validation (k = 10) yielded a mean R^2^ of 0.019 ± 0.376 (fold-wise R^2^: −0.433, −0.676, −0.121, 0.474, 0.421, −0.377, 0.237, 0.142, 0.217, 0.304), a mean MAE of 0.738 ± 0.221 Hz, and a mean RMSE of 0.918 ± 0.306 Hz. The near-zero mean R^2^ and the substantial variability across folds (four negative values; range: −0.68 to +0.47) can be attributed to the small number of subjects per validation subset (~8 per fold), which renders fold-specific R^2^ estimates highly sensitive to the age composition of each subset. When the between-subject age variance within a fold is limited, the denominator of R^2^ (total sum of squares) is dominated by residual (non-age) variance, producing near-zero or negative R^2^ values even when the underlying age–iAPF association is genuine. A permutation test confirmed that the observed 10-fold mean R^2^ was significantly above chance (*p* = 0.001), indicating that the low mean R^2^ reflects instability of the metric rather than absence of predictive signal.

To obtain a more stable estimate of generalization performance, leave-one-subject-out cross-validation was conducted. LOOCV yielded an R^2^ of 0.155, an MAE of 0.744 Hz, and an RMSE of 0.972 Hz. The LOOCV slope estimate was highly stable across all 81 iterations (mean = −0.054 Hz/year; SD = 0.001; range: −0.059 to −0.049; coefficient of variation = 2.6%). A permutation test confirmed that the LOOCV R^2^ was significantly above chance (*p* < 0.001). The LOOCV R^2^ (0.155) was lower than the full-sample R^2^ (0.198), indicating some optimism in the in-sample estimate, but remained substantially above zero and stable across all iterations. Taken together, these results indicate that the age-only model has limited but non-negligible predictive performance on unseen data and that the instability of 10-fold R^2^ values is attributable to the small fold size rather than to absence of an age–iAPF association. Bootstrap resampling (1000 iterations) produced the following 95% confidence intervals: intercept β_0_ = 13.22 [11.46, 15.16] Hz, slope β_1_ = −0.054 [−0.082, −0.030] Hz/year, and R^2^ = 0.198 [0.067, 0.368] (Table 2). The bootstrap confidence interval for the slope excludes zero, confirming that the age–iAPF association is statistically reliable, while the cross-validation results indicate that predictive performance on unseen data, though above chance, remains limited.

Influence diagnostics using Cook’s distance identified four cases with D > 0.049 (threshold = 4/81), representing 4.9% of the sample. These cases comprised one participant with a high iAPF (age = 60, iAPF = 13.957 Hz; D = 0.206; studentized residual = +4.22) and three participants with relatively low iAPF values for their age (age = 83, iAPF = 7.188 Hz; age = 57, iAPF = 7.761 Hz; age = 55, iAPF = 8.601 Hz; D = 0.069, 0.103, and 0.062, respectively). A sensitivity analysis excluding all four influential cases yielded a revised model (iAPF = 13.14 − 0.053 × Age; R^2^ = 0.251; F(1,75) = 25.11, *p* < 0.001), in which the slope changed by only 2.5% relative to the full model. In addition, robust regression using Huber (slope = −0.052 Hz/year, *p* < 0.001) and Tukey biweight (slope = −0.050 Hz/year, *p* < 0.001) estimators—which down-weight influential observations rather than excluding them—yielded slope estimates within the same range. Across all sensitivity approaches, the age slope remained between −0.049 and −0.054 Hz/year, was consistently negative and statistically significant (all *p* < 0.001), and fell within the 95% bootstrap confidence interval of the full-model slope [−0.082, −0.030].

To further evaluate whether the highest iAPF value (13.957 Hz, located 0.043 Hz from the upper search boundary of 14 Hz) might represent a boundary-related artifact, a sensitivity analysis was conducted using a narrower alpha search range (8–13 Hz). Only this single participant would have been excluded under the narrower range. The resulting model (iAPF = 12.80 − 0.049 × Age; R^2^ = 0.204; *p* < 0.001) confirmed that the slope estimate remains stable. Additionally, the FOOOF goodness-of-fit for this participant was R^2^ = 0.853, above the acceptable threshold of 0.80, with five periodic peaks detected and the alpha peak correctly identified above the fitted aperiodic component. Visual inspection of the individual FOOOF spectral decomposition confirmed that the 13.957 Hz peak corresponded to a genuine oscillatory peak rather than a boundary artifact or spectral parameterization error. Given that this participant met all inclusion criteria (cognitively healthy, MMSE = 30, no neurological or psychiatric history) and that all sensitivity analyses converged on a stable slope estimate, the case was retained in the primary model to preserve sample representativeness.

### 3.4. FOOOF Goodness-of-Fit and Exploratory Analysis of Aperiodic Parameters

The FOOOF algorithm yielded excellent spectral fits across all 81 participants (mean R^2^ = 0.936 ± 0.079; median = 0.961; 5th–95th percentile range: 0.853–0.981). Eight participants had R^2^ values below 0.90, and three had R^2^ values below 0.80 (0.49, 0.70, and 0.74). These three participants were retained after visual inspection of their spectral decompositions confirmed that the alpha peak was correctly identified above the fitted aperiodic component. No participants were excluded due to poor spectral fit. The mean fitting error was 0.068 ± 0.017 (range: 0.041–0.141). The mean number of periodic peaks detected per participant was 4.93 ± 1.06 (range: 2–6). The mean alpha peak amplitude was 1.178 ± 0.477, and the mean alpha peak bandwidth (FWHM) was 1.657 ± 0.811 Hz. The mean number of retained 2 s epochs was 115.4 ± 16.0 (range: 76–151; mean recording duration: 3.8 min), with six posterior channels available per participant. The mean aperiodic exponent was 0.711 ± 0.367 (range −0.31 to 1.62), and the mean aperiodic offset was −11.628 ± 0.466.

The aperiodic exponent was not significantly correlated with age (r = −0.121, *p* = 0.280), nor was the aperiodic offset (r = −0.134, *p* = 0.234). However, the aperiodic exponent showed a significant negative association with iAPF (r = −0.299, *p* = 0.007), which remained significant after controlling for age (partial r = −0.397, *p* = 0.0002), indicating that participants with a flatter spectral slope tended to show lower iAPF values independently of age. An exploratory multiple regression including both age and the aperiodic exponent as predictors yielded R^2^ = 0.325 (adjusted R^2^ = 0.307; AIC = 213.21), compared to R^2^ = 0.198 for the primary age-only model (ΔAIC = −11.89), with the exponent contributing significantly (β = −1.038, 95% CI [−1.580, −0.497], *p* = 0.0003). These findings are reported as exploratory and do not modify the primary age-adjusted model, which retains age as the sole predictor.

### 3.5. Comparison of FOOOF-Derived and Conventional PSD-Based iAPF Estimates

To evaluate the methodological advantage of spectral parameterization over conventional peak detection, the iAPF was estimated using two approaches: (a) FOOOF-derived alpha peak (iAPF_FOOOF), defined as the center frequency of the highest-amplitude periodic peak within 7–14 Hz after aperiodic separation, and (b) conventional PSD peak (iAPF_PSD), defined as the frequency of maximum power within 7–14 Hz on the raw, unparameterized PSD. Both estimates were derived from the same Welch PSDs, ensuring that any differences reflect the parameterization step rather than spectral estimation differences.

The two methods showed a moderate correlation (r = 0.707, *p* < 0.001) but notable discrepancies at the individual level. The mean absolute difference was 0.377 ± 0.719 Hz (range: 0–4.04 Hz), with 16.0% of participants differing by more than 0.5 Hz and 9.9% by more than 1.0 Hz. A paired-samples t-test did not reach statistical significance (t(80) = 1.88, *p* = 0.064), and Bland–Altman analysis showed a bias of 0.166 Hz with limits of agreement from −1.39 to 1.73 Hz.

Critically, the two methods differed substantially in their sensitivity to age. The FOOOF-derived iAPF showed a significant negative association with age (slope = −0.054 Hz/year, R^2^ = 0.198, *p* < 0.001), whereas the conventional PSD-based iAPF showed a substantially weaker and only marginally significant relationship (slope = −0.027 Hz/year, R^2^ = 0.055, *p* = 0.035). This approximately threefold difference in explained variance suggests that the aperiodic 1/f background confounds conventional peak detection in older adults, attenuating the apparent age–iAPF relationship. These results directly support the use of FOOOF-based spectral parameterization for estimating alpha center frequency in aging.

### 3.6. Exploratory Multiple Regression: Effect of Sex

To formally address the potential confounding role of sex, a multiple regression model was fitted including both age and sex as predictors (Model 2: iAPF ~ Age + Sex). Sex was not a significant predictor of iAPF (β = 0.003 Hz, 95% CI [−0.437, 0.443]; *p* = 0.989), its inclusion produced no change in R^2^ (ΔR^2^ = 0.000), and the age slope remained identical to the age-only model (β = −0.054 Hz/year; change = 0.0%). The Age × Sex interaction was also non-significant (*p* = 0.992), indicating that the rate of age-related iAPF decline does not differ between sexes. Model fit indices confirmed that the age-only model is preferable (ΔAIC = +2.0; ΔBIC = +4.4 upon adding sex). These results are reported in Table 4.

### 3.7. Exploratory Regression: Effect of MMSE and Education

Given that the MMSE was used as an exclusion criterion (≥24) rather than a covariate, its potential predictive contribution to iAPF was examined exploratorily. MMSE scores ranged from 24 to 30 (M = 29.05, SD = 1.21), but the distribution was strongly negatively skewed (skewness = −1.82; Shapiro–Wilk W = 0.753, *p* < 0.001), with 45.7% of participants scoring the ceiling value (30/30) and 75.3% scoring ≥ 29. MMSE was not significantly correlated with iAPF (r = +0.159, *p* = 0.156). In a multiple regression including both age and MMSE, the overall model explained 20.4% of iAPF variance (R^2^ = 0.204; adjusted R^2^ = 0.184), a negligible improvement over the age-only model (ΔR^2^ = 0.006). MMSE was not a significant predictor (β = 0.069, *p* = 0.447), and model parsimony worsened (ΔAIC = +1.40; ΔBIC = +2.79). These results confirm that the MMSE ceiling effect precludes its utility as a covariate in the present age-adjusted model.

To evaluate the potential predictive value of educational attainment, an exploratory regression model was fitted including both age and education as predictors. Education showed a significant positive association with iAPF (r = +0.267, *p* = 0.016), which remained significant after controlling for age (partial r = +0.290, *p* = 0.009). The multiple regression model yielded R^2^ = 0.266 (adjusted R^2^ = 0.247; AIC = 219.97), compared to R^2^ = 0.198 for the age-only model (ΔAIC = −5.13), with education contributing significantly (β = 0.067, 95% CI [0.017, 0.117]; *p* = 0.009). These findings suggest that higher educational attainment is associated with higher iAPF independently of age, consistent with cognitive reserve frameworks. These results are reported as exploratory and do not modify the primary aged-adjusted model; the age-only model is retained in accordance with the original study design.

### 3.8. Age-Adjusted Reference Range

A progressive decrease in mean iAPF was observed across age groups (Table 5), with an overall reduction of approximately 0.9 Hz between the youngest group (50–59 years) and the oldest group (80–89 years), with relative stabilization in the ninth decade. In addition to the observed range, Table 5 includes an age-adjusted variability band (±1 SD) derived from the regression model using the midpoint of each age interval. This band describes expected within-age-group variability and is not intended as a clinical reference range.

### 3.9. Residual Analysis

**Figure 4:**
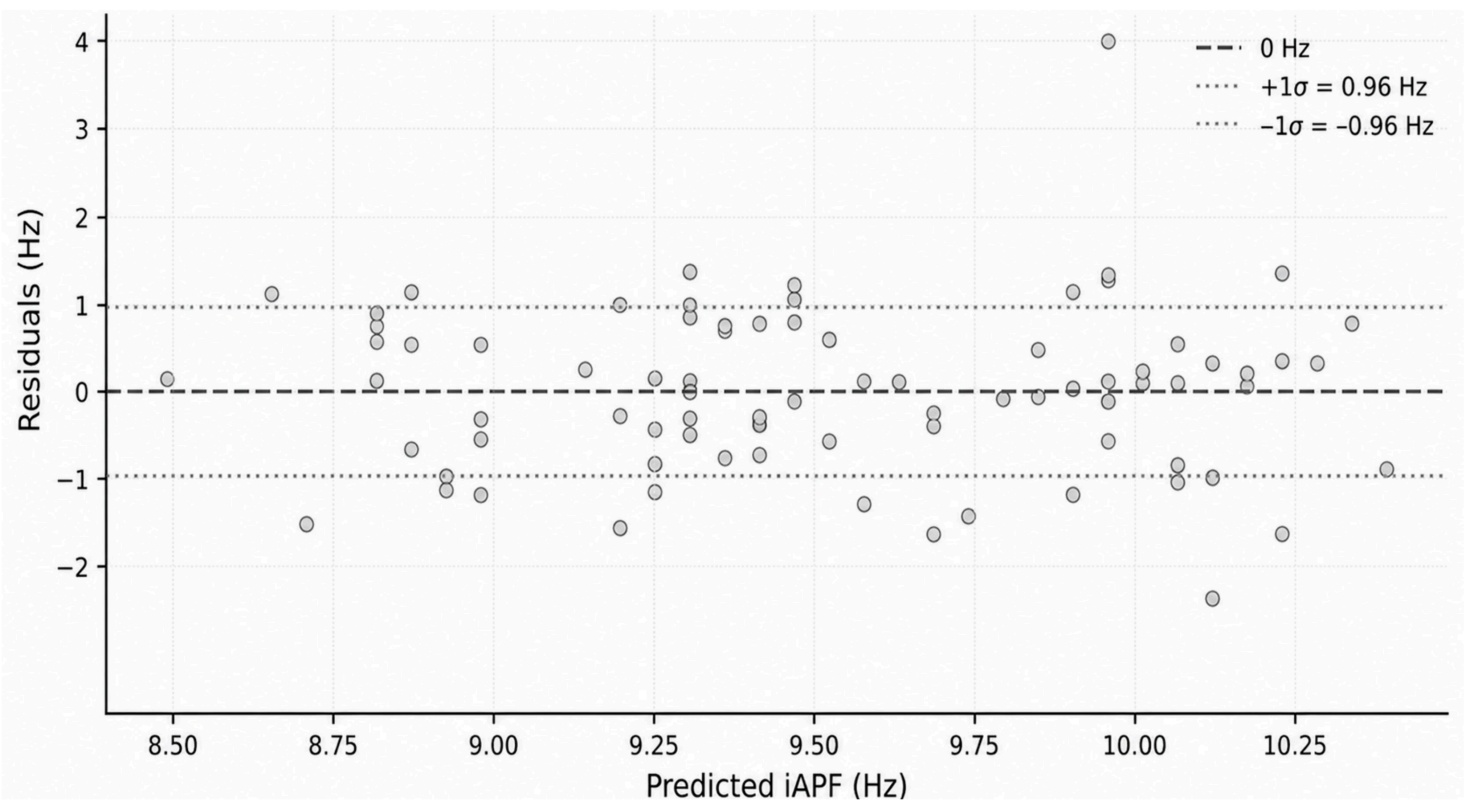
Scatterplot of residuals against predicted iAPF values. The central dashed line indicates zero residual error, whereas the dotted lines indicate ±1 residual standard deviation (±0.96 Hz).

**Figure 5:**
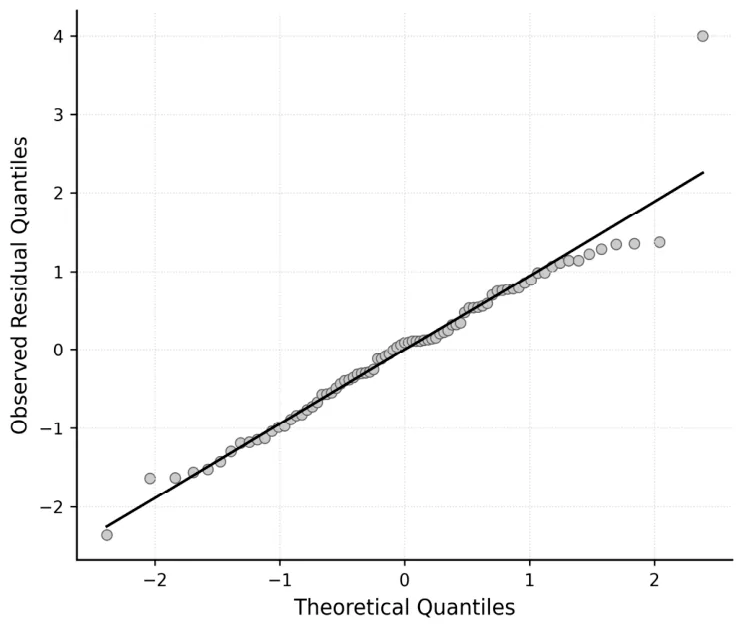
Q–Q plot comparing the standardized residual distribution with the theoretical normal distribution. Most points follow the reference line, with deviations mainly in the tails.

Residual analysis (Figure 4 and Figure 5) suggested no major violations of model assumptions. The Shapiro–Wilk test was significant (W = 0.9555, *p* = 0.0067), suggesting a mild departure from normality. However, visual inspection of the Q–Q plot showed that most residuals followed the theoretical reference line, with deviations mainly in the tails. Given the sample size (n = 81), the linear regression model can be considered reasonably robust to moderate deviations from normality.

### 3.10. Exploratory Age-Adjusted Reference Band

A wider exploratory age-adjusted variability band was defined to describe the expected range of iAPF values at a given age. It should be noted that this band was calculated as predicted value ± 1.96 residual standard deviations, which approximates but does not equal a formal 95% prediction interval. A proper regression prediction interval incorporates uncertainty in the estimated regression coefficients and therefore widens toward the extremes of the predictor range. At the mean age (67.9 years), the approximate 95% prediction interval is ±1.92 Hz, whereas at ages 55 and 85 years, it widens to ±1.95 and ±1.97 Hz, respectively. Accordingly, the band ±1.88 Hz used here represents a simplification that slightly underestimates the true prediction uncertainty, particularly at the extremes of the age range. This exploratory band is reported for descriptive purposes only and is not intended as a clinical reference range or screening cutoff.

## 4. Discussion

This study developed an age-adjusted model of iAPF in healthy older adults using FOOOF-based spectral parameterization.

The negative slope observed in our model (−0.054 Hz/year, equivalent to a decrease of 0.54 Hz per decade) is consistent with the previous literature documenting a progressive slowing of the alpha rhythm with aging (Grandy et al., 2013; Scally et al., 2018). This decline is consistent with the literature linking alpha slowing to neurobiological changes associated with aging, including alterations in thalamocortical circuits (Hughes & Crunelli, 2005), a possible reduction in cholinergic modulation (Chaves-Coira et al., 2023; Hindriks & Van Putten, 2013) and changes in neural synchronization (Pascarella et al., 2025; Scally et al., 2018), although the present study does not allow the specific contribution of these mechanisms to be established. In this sense, Grandy et al. (2013) reported an approximately 1 Hz reduction in iAPF between young adults (20–30 years) and older adults (60–70 years), which corresponds to roughly 0.2–0.25 Hz per decade. Although this rate is lower than that observed in our study, this difference may be explained by the fact that our sample is concentrated in individuals over 50 years of age, a period in which iAPF decline may be more pronounced (Turner et al., 2023). Consistently, Köpruner et al. (1984), using a regression-based approach, also reported a decrease in iAPF with age, with an approximate slope of 0.053 Hz/year within the alpha range (11.95 − 0.053 × Age), which further supports the general direction of our findings. Although we observed a similar slope, the intercept in our model was higher (13.22 vs. 11.95 reported by those authors), possibly because we removed the aperiodic component before estimating iAPF. Moreover, the R^2^ value of 0.198 is reasonable, given that iAPF is not determined solely by age but is also influenced by multiple biological and individual factors, including genetic influences (Smit et al., 2006), cognitive functioning (Corcoran et al., 2018; Grandy et al., 2013), and overall brain health (De Frutos-Lucas et al., 2018; May et al., 2025). In this sense, the interindividual variability observed in our sample does not diminish the utility of the model but rather underscores the multifactorial nature of iAPF and the value of interpreting it within a broader clinical and neurobiological framework (Finley et al., 2024). Furthermore, although the explanatory capacity of the model was moderate, bootstrap resampling confirmed that the age–iAPF association was statistically reliable, with the 95% confidence interval for the slope excluding zero [−0.082, −0.030]. This statistical reliability of the association should be distinguished from predictive performance: 10-fold cross-validation indicated limited and unstable predictive accuracy on held-out data (mean R^2^ = 0.019, with multiple negative fold-specific values), largely attributable to the small number of subjects per validation subset (~8) and the resulting instability of fold-specific variance estimates. Leave-one-subject-out cross-validation provided a more stable estimate (R^2^ = 0.155; permutation *p* < 0.001), confirming that the model retains limited but non-negligible predictive value on unseen data, while the gap between LOOCV R^2^ (0.155) and full-sample R^2^ (0.198) indicates some in-sample optimism. These results are consistent with the moderate variance explained by the full model and reflect the substantial interindividual variability in iAPF that is not captured by age alone.

Taken together, these findings are consistent with a broadly linear decline in iAPF across the 52–87 year age range examined. Although the possibility of accelerated slowing in later stages of aging has been suggested in the literature, the present analyses did not find clear statistical evidence for a nonlinear age–iAPF relationship, as quadratic, piecewise, and spline models did not significantly improve fit over the linear specification. Future studies with broader age ranges and larger samples may be better positioned to detect potential nonlinearities if they exist.

From a methodological standpoint, one of the main contributions of the present study lies in the estimation of iAPF through FOOOF-based spectral parameterization. As previous work has pointed out, EEG analyses relying exclusively on canonical frequency bands may conflate changes in oscillatory peaks with variations in the aperiodic component of the spectrum, thereby compromising the physiological specificity of the interpretation (Tröndle et al., 2023). In older populations, this limitation becomes particularly important, as slowing of the alpha rhythm may shift the dominant activity outside the classical boundaries of the 8–12 Hz band. Within this framework, the separation of periodic and aperiodic components allows for a more precise estimation of alpha center frequency and reduces the risk of bias arising from rigid frequency-based definitions (Donoghue et al., 2020; Leroy et al., 2025). Therefore, the model proposed here provides a preliminary quantification of the age–iAPF relationship using an analytical strategy that is better aligned with the spectral complexity of EEG in aging, thereby increasing the physiological specificity of its interpretation.

A further consideration concerns the aperiodic component of the EEG spectrum, which itself undergoes significant age-related changes. It is well established that the aperiodic 1/f exponent decreases with age, reflecting a flattening of the spectral slope that has been interpreted as increased neural noise or reduced cortical inhibition (Merkin et al., 2023; Tröndle et al., 2023). These changes in the aperiodic background may partly confound the interpretation of age-related alpha slowing when conventional spectral analyses are used, as shifts in the aperiodic component can alter the apparent amplitude and frequency of oscillatory peaks. The spectral parameterization approach employed in the present study mitigates this concern by explicitly separating the aperiodic and periodic components before estimating the iAPF. In the present study, the aperiodic exponent and offset were not included as primary predictors, as the study was designed to establish an age-only preliminary framework. However, exploratory analyses revealed a significant association between the aperiodic exponent and iAPF that persisted after controlling for age (partial r = −0.397, *p* = 0.0002), and a multiple regression including both age and the aperiodic exponent explained 32.5% of iAPF variance (adjusted R^2^ = 0.307), compared to 19.8% for the age-only model. Notably, the aperiodic exponent was not significantly correlated with age in the present sample (r = −0.121, *p* = 0.280), which may reflect the restricted age range or sample size. These findings motivate future studies that systematically incorporate aperiodic parameters alongside periodic component (Merkin et al., 2023; Tröndle et al., 2023).

From a neurophysiological standpoint, the present model characterizes the population-level trajectory of iAPF decline across healthy aging, providing an age-calibrated reference for contextualizing alpha slowing at the group level. The observed negative age–iAPF association (β = −0.054 Hz/year), adequately described by a linear specification within the examined age range, is consistent with previous studies and reflects the progressive neurobiological changes associated with aging, including alterations in thalamocortical circuits and cortical synchronization. However, the model’s utility is strictly limited to group-level neurophysiological description: the 95% prediction interval at the individual level is approximately ±1.92 Hz (spanning approx. 3.84 Hz in total), which precludes any meaningful application at the single-subject level. No claims of individual-level screening or clinical diagnostic utility are therefore made. Future models incorporating additional covariates and larger samples may achieve the precision required for individual-level interpretation.

This view is also consistent with the literature that conceptualizes iAPF as a relatively stable marker, with good intra-individual and test–retest reliability in both younger and older adults (Popov et al., 2023). That stability does not imply that the parameter remains unchanged in the face of aging; rather, it reinforces its relevance as a reference measure for characterizing interindividual differences and trajectories of change. In other words, the utility of iAPF lies not in its static nature but in the fact that it combines metric consistency with sensitivity to neurophysiologically relevant processes of brain aging. This dual property further strengthens the rationale for using it as a target variable in age-adjusted models such as the one proposed in the present study.

Overall, the present findings should be interpreted within the limits of the study design. The model was derived from eyes-closed resting-state EEG recordings and posterior electrodes, and therefore, its generalization to other experimental conditions, activation states, or topographical configurations cannot be assumed without further evidence. Although the age–iAPF association was statistically reliable (bootstrap 95% CI for the slope excluding zero) and LOOCV confirmed limited but significant predictive performance (R^2^ = 0.155, permutation *p* < 0.001), age accounted for only a moderate proportion of the total variance and 10-fold cross-validation revealed substantial instability in fold-specific estimates, indicating that other biological, cognitive, or contextual factors make important contributions to interindividual variability. Accordingly, age should be understood as a relevant, but not exhaustive, determinant of iAPF. The model estimates should be regarded as preliminary until externally validated in independent cohorts. Future studies should prioritize the evaluation of this framework in larger samples and, once prediction uncertainty is reduced through the incorporation of additional covariates, examine its potential translational value in populations at risk of neurodegenerative decline.

Furthermore, with 81 participants distributed across a 35-year age range, the average sampling density amounts to approximately 2–3 individuals per age stratum, which limits the resolution of the model estimates, particularly at the extremes of the age range. This limitation underscores the importance of the sensitivity analyses reported above and highlights the need for larger cohorts in future studies.

Although sex was not included as a covariate in the final model, formal testing confirmed it was not a significant predictor of iAPF (see Section 3.5). This finding is consistent with evidence that sex differences in iAPF are mediated by hormonal fluctuations of the menstrual cycle (Bazanova & Vernon, 2014) that are absent in a fully postmenopausal cohort (age range 52–87 years; (González Alonso, 2021)). Future studies spanning pre- and postmenopausal ages should explicitly model sex and hormonal status.

Exploratory analyses revealed that educational attainment was a significant positive predictor of iAPF, independent of age (see Section 3.6). This finding indicates that education carries explanatory value beyond age and should be systematically incorporated in future models, particularly in samples with wide educational ranges.

Cognitive status was not included as a covariate because the MMSE was used solely as an exclusion criterion (≥24), producing a pronounced ceiling effect (45.7% scoring 30/30; see Section 3.7). Empirical testing confirmed that MMSE was not a significant predictor of iAPF in this sample. Future studies should employ more sensitive cognitive instruments (e.g., MoCA, RBANS) capable of discriminating within the upper range of cognitive performance.

Additionally, influence diagnostics identified four influential cases (Cook’s D > 4/n). Although the slope estimate showed a 10% change when the single most influential case (iAPF = 13.957 Hz) was excluded, excluding all four influential cases simultaneously changed the slope by only 2.5%, and robust regression (Huber and Tukey) yielded slopes within the same range (−0.050 to −0.052 Hz/year). These results confirm that the age–iAPF association is not driven by individual observations. Nevertheless, the presence of influential cases underscores the vulnerability of regression estimates in modestly sized samples and highlights the importance of larger cohorts.

Moreover, the considerable interindividual variability in iAPF (SD = 1.064 Hz) and the moderate R^2^ result in relatively wide prediction intervals (approximately ±1.88 Hz at the 95% level), which constrain the model’s sensitivity for detecting individual-level deviations. Consequently, the model should be regarded exclusively as a group-level neurophysiological reference characterizing healthy aging trajectories and must not be interpreted as having individual-level screening or diagnostic sensitivity.

Taken together, the present study provides a preliminary age-adjusted model of individual alpha peak frequency in healthy older adults, obtained through a spectral parameterization approach that improves the specificity of the estimate. Rather than proposing a diagnostic criterion or clinical reference framework, our findings offer a preliminary age-calibrated equation for describing alpha slowing across healthy aging at the group level. From this perspective, the model may provide a useful foundation for future research on group-level neurophysiological aging trajectories, while individual-level clinical applications must await externally validated models with substantially reduced prediction uncertainty.

## 5. Conclusions

In summary, this study provides a preliminary age-adjusted model of individual alpha peak frequency in cognitively healthy older adults based on FOOOF-derived spectral parameterization of resting-state EEG. Across participants aged 52 to 87 years, iAPF showed a significant linear decrease with age, described by the equation iAPF = 13.22 − 0.054 × age, corresponding to an average decline of approximately 0.54 Hz per decade. Although age accounted for a modest proportion of the total variance, the age–iAPF association was statistically reliable (bootstrap 95% CI excluding zero), even though cross-validation indicated limited predictive performance on unseen data (10-fold R^2^ = 0.019; LOOCV R^2^ = 0.155). The model offers a descriptive reference equation for characterizing iAPF trajectories at the group level in later life. By separating periodic alpha activity from the aperiodic background, the present approach avoids an important limitation of conventional band-based EEG analyses and provides a physiologically more specific estimate of peak alpha dynamics. In this sense, the proposed model describes population-level iAPF trajectories across healthy aging but does not possess sufficient precision for individual-level clinical application, given a 95% prediction interval of approximately ±1.92 Hz, while reducing interpretive bias associated with fixed canonical frequency bands (Donoghue et al., 2020; Scally et al., 2018).

Future studies should test the external validity of this model in independent cohorts, examine the contribution of additional covariates (e.g., sex, education, cognitive status), and evaluate whether age-adjusted iAPF models can eventually achieve the precision required for meaningful clinical application in populations at risk of cognitive decline.

## Figures and Tables

**Table 1:** Sample characteristics.

Variable	M (SD)	Range
Age, years	67.93 (8.72)	52–87
Education, years	12.64 (4.11)	1–21
MMSE, total score	29.05 (1.21)	24–30
MMSEc	28.07 (1.47)	24.4–30.0
Sex, n (%)		
— Women	49 (60.5%)	—
— Men	32 (39.5%)	—

Note. MMSE = Mini-Mental State Examination; MMSEc = education-corrected MMSE.

**Table 2:** Linear Regression Model Parameters.

Parameter	Estimate	95% CI	*p*
Intercept (β_0_), Hz	13.216	[11.46, 15.16]	<0.001
Slope (β_1_), Hz/year	−0.0543	[−0.082, −0.030]	<0.001
R^2^	0.198	[0.067, 0.368]	<0.001
Adjusted R^2^	0.188	—	—
MAE, Hz	0.725	—	—
RMSE, Hz	0.947	—	—
Residual SD (σ), Hz	0.960	—	—

Note. CI = 95% confidence interval estimated via bootstrapping (1000 iterations). MAE = mean absolute error; RMSE = root mean square error; SD = standard deviation.

**Table 3:** Comparison of Regression Models for the Age–iAPF Relationship.

Model	k	AIC	BIC	R^2^	Adj. R^2^	ΔAIC	LRT *p*
Linear	2	225.10	229.89	0.198	0.188	—	—
Quadratic	3	227.09	234.28	0.198	0.178	+1.99	—
Piecewise (B*P* = 60, fixed)	3	226.40	233.58	0.205	0.185	+1.30	0.402
Piecewise (B*P* = 60, data-driven)	3	226.40	233.58	0.205	0.185	+1.30	0.402
RCS (3 knots)	3	227.10	234.28	0.198	0.178	+2.00	0.951
RCS (4 knots)	4	228.82	238.39	0.201	0.170	+3.72	—

Note. k = number of parameters; AIC = Akaike Information Criterion; BIC = Bayesian Information Criterion; R^2^ = coefficient of determination; Adj. R^2^ = adjusted R^2^; ΔAIC = change in AIC relative to the linear model; LRT *p* = *p*-value from the likelihood ratio test comparing each model to the linear model. For the piecewise model, a grid search across the age range (55–84 years) identified 60 years as the data-driven optimal breakpoint. All ΔAIC values are <4, indicating that no alternative model demonstrated clear evidence of improved fit over the linear specification (Burnham & Anderson, 2002). The linear model was retained on the basis of parsimony.

**Table 4:** Comparison of Linear Regression Models for iAPF: Age-Only vs. Age + Sex.

Model	R^2^	Adjusted R^2^	AIC	BIC
iAPF − Age	0.198	0.188	225.10	229.89
iAPF − Age + Sex	0.198	0.177	227.10	234.28

Note. β = unstandardized regression coefficient; CI = 95% confidence interval estimated via bootstrapping (1000 iterations); SE = standard error; t = t-statistic; *p* = two-tailed p-value; R^2^ = coefficient of determination; Adj. R^2^ = adjusted R^2^; AIC = Akaike Information Criterion; BIC = Bayesian Information Criterion; ΔAIC and ΔBIC = change in AIC and BIC relative to Model 1 (Age-only). Sex was coded as a binary dummy variable (female = 1, male = 0). The Age × Sex interaction term was tested in a separate model and was non-significant (*p* = 0.992). Reference category for sex: male. Model 1 is retained as the final model on the basis of lower AIC, higher adjusted R^2^, and the non-significance of the sex predictor.

**Table 5:** Age-adjusted variability band (±1 SD).

Age Range (Years)	n	iAPF Mean ± SD (Hz)	Range	Age-Adjusted Reference Band (Hz)
50–59	17	9.959 ± 0.961	7.76–11.58	9.32–11.24
60–69	25	9.922 ± 1.208	8.05–13.96	8.78–10.70
70–79	29	9.088 ± 0.833	7.64–10.68	8.25–10.16
80–89	10	9.081 ± 0.866	7.19–10.01	7.70–9.62

Note. Variability band estimated from the regression model using the midpoint of each age interval and a residual standard deviation of ±0.96 Hz. This band describes the expected within-age-group variability (±1 SD) and does not represent a clinical reference range or prediction interval.

## Data Availability

The data analyzed in this study were obtained through the eBRAIN-Health platform within the eBRAIN-Health project (HORIZON-INFRA-2021-TECH-01-01; Grant Agreement No. 101058516), using the PDWAVES database metafile (DOI: 10.25493/8MVP-NPN; DOI: 10.25493/FWHQ-6W5). The authors do not own these data and therefore cannot redistribute them. Requests for access to the underlying dataset should be directed to the originating program and the corresponding data custodians through the eBRAIN-Health platform.

## Abbreviations

The following abbreviations are used in this manuscript:

EEG	Electroencephalogram
AD	Alzheimer’s Disease
FOOOF	Fitting Oscillations and One-Over-F
Hz	Hertz
